# Isolation of full-length IgG antibodies from combinatorial libraries expressed in the cytoplasm of *Escherichia coli*

**DOI:** 10.1038/s41467-023-39178-x

**Published:** 2023-06-14

**Authors:** Michael-Paul Robinson, Jinjoo Jung, Natalia Lopez-Barbosa, Matthew Chang, Mingji Li, Thapakorn Jaroentomeechai, Emily C. Cox, Xiaolu Zheng, Mehmet Berkmen, Matthew P. DeLisa

**Affiliations:** 1 Robert F. Smith School of Chemical and Biomolecular Engineering, Cornell University, Ithaca, NY 14853 USA; 2 Biomedical and Biological Sciences, College of Veterinary Medicine, Cornell University, Ithaca, NY 14853 USA; 3 New England Biolabs, 240 County Road, Ipswich, MA 01938 USA; 4 Cornell Institute of Biotechnology, Cornell University, Ithaca, NY 14853 USA

**Keywords:** Applied immunology, Expression systems, Protein design, Combinatorial libraries, Synthetic biology

## Abstract

Here we describe a facile and robust genetic selection for isolating full-length IgG antibodies from combinatorial libraries expressed in the cytoplasm of redox-engineered *Escherichia coli* cells. The method is based on the transport of a bifunctional substrate comprised of an antigen fused to chloramphenicol acetyltransferase, which allows positive selection of bacterial cells co-expressing cytoplasmic IgGs called cyclonals that specifically capture the chimeric antigen and sequester the antibiotic resistance marker in the cytoplasm. The utility of this approach is first demonstrated by isolating affinity-matured cyclonal variants that specifically bind their cognate antigen, the leucine zipper domain of a yeast transcriptional activator, with subnanomolar affinities, which represent a ~20-fold improvement over the parental IgG. We then use the genetic assay to discover antigen-specific cyclonals from a naïve human antibody repertoire, leading to the identification of lead IgG candidates with affinity and specificity for an influenza hemagglutinin-derived peptide antigen.

## Abstract (web summary)

Discovery of full-length antibodies can be slow and labor intensive. Here, the authors describe a robust genetic assay for facile isolation of IgG antibodies from combinatorial libraries expressed in the cytoplasm of redox-engineered bacteria.

## Introduction

Monoclonal antibodies (mAbs) represent one of the fastest-growing segments of the biotechnology industry, enabling dramatic advances in biomedical research and modern medicine. Accordingly, technologies capable of straightforward identification and molecular engineering of full-length immunoglobulin G (IgG) antibodies are in great demand. Conventional procedures for isolating mAbs include various hybridoma technologies that involve immunization followed by cell fusion^1–3^ and protein engineering platforms such as phage display^4^, ribosome and mRNA display^5^, and microbial cell display technologies^6–9^ that permit high-throughput screening of large recombinant antibody libraries. Hybridoma-based methods for isolating antibodies are labor- and time-intensive, incompatible with multiplexing and parallelization, and do not permit customization of mAb properties such as antigen-binding affinity, stability, or expression level. These shortcomings can be circumvented by the use of display technologies; however, these methods are typically built around libraries of smaller, more conveniently expressed derivatives of mAbs such as single-chain variable antibody fragments (scFv) or antigen-binding fragments (Fabs)^8^. Compared to their IgG counterparts, these smaller formats often exhibit weaker monovalent binding and poor serum persistence in animals, the latter of which stems from their relatively low molecular weight and lack of an Fc domain. Consequently, antibody fragments isolated using display technologies require molecular conversion to IgG format prior to therapeutic development.

More recently, cell surface display of full-length IgGs has been demonstrated in bacteria^10–12^, yeast^13–15^, and mammalian cells^16^, effectively circumventing the reformatting issue. Screening methods such as these require each library member to be individually evaluated, which necessitates (i) specialized equipment (e.g., flow cytometer) to access meaningful amounts of sequence space and (ii) a high-quality screening antigen, typically a recombinant protein that must be separately purified and fluorescently labeled. It should be noted that even with state-of-the-art instrumentation, the screening of combinatorial libraries with diversity >10^8^ is technically challenging^12,17^. Another drawback of cell surface display is the inherent bias and complexity that can be introduced by the need for energetically unfavorable trafficking of IgG molecules across one or more biological membranes, which are known to selectively eliminate clones that are unfit for translocation but might otherwise be viable. Moreover, IgG display in yeast cells involves a secretion-capture process that is prone to crosstalk among library members while in mammalian cells the process suffers from limited library sizes due to low transfection efficiency and the appearance of multiple copies of antibodies with different specificities on a single cell surface, making it difficult to directly identify and isolate antibodies with desired properties from naïve libraries.

To address these shortcomings, here we describe a genetic selection strategy for isolating full-length IgGs from combinatorial libraries expressed in the cytoplasm of *Escherichia coli*. Specifically, the method leverages bifunctional substrate proteins comprised of an antigen fused to chloramphenicol acetyltransferase (CAT) whose translocation through the twin-arginine translocation (Tat) pathway^18^ is toggled by the presence or absence of a cytoplasmically co-expressed IgG antibody known as a cyclonal^19^. In this manner, capture of the chimeric antigen by a co-expressed IgG effectively sequesters the CAT antibiotic resistance marker in the cytoplasm, catalyzing detoxification of chloramphenicol and permitting positive selection for antigen binding. By using the genetically engineered *E. coli* strain SHuffle, which promotes efficient cytoplasmic disulfide bond formation^20^, it is possible to achieve high-level functional expression of cyclonals within the cytoplasmic compartment while at the same time bypassing the need for membrane translocation of IgG molecules. Moreover, compared with screening methods that necessitate analysis of each individual IgG variant, our selection directly eliminates unwanted IgG variants through the application of tunable selective pressure on the mutant library. This feature of selection makes it intrinsically high throughput, enabling interrogation in theory of very large libraries (>10^11^). We first demonstrated the utility of this approach by isolating cyclonals with unique complementarity-determining regions (CDRs) that promote specific binding to the basic-region leucine zipper domain of the yeast transcriptional activator Gcn4. We then used the genetic assay for discovering antigen-specific lead IgG candidates from a naïve human antibody repertoire, leading to the identification of cyclonals with human CDRs that bind specifically to an influenza hemagglutinin (HAG) peptide antigen. Importantly, discovery of these different cyclonals was made possible by simply demanding bacterial growth on defined concentrations of antibiotic, obviating the need for purification, labeling or immobilization of the target antigen. Hence, our selection represents a straightforward tool for enrichment of productive binders in the IgG format and offers a compelling alternative to conventional methods that are more expensive, time-consuming, and labor-intensive.

## Results

### Design of a positive selection for antigen-binding activity in the cytoplasm

**Fig. 1:**
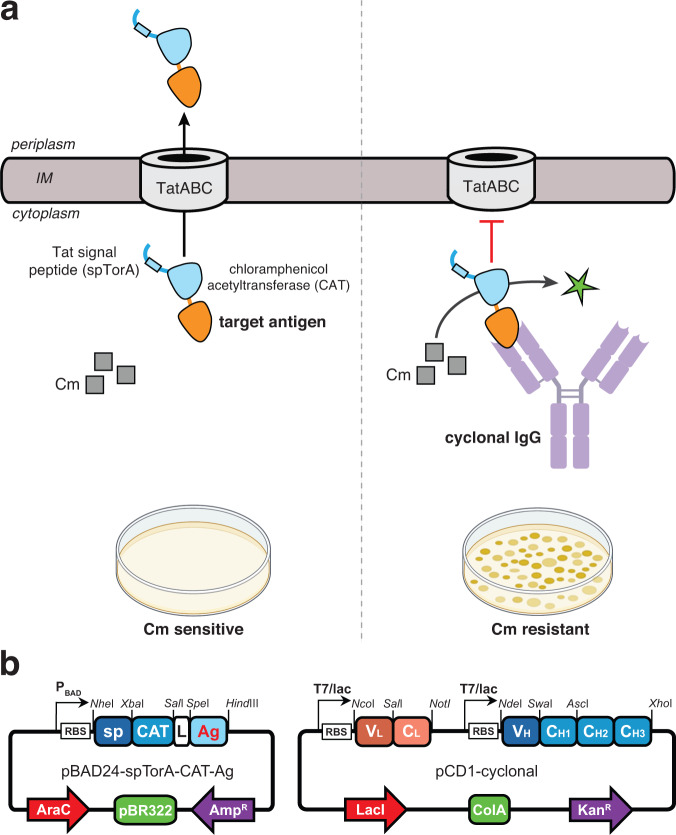
Genetic selection for isolating full-length IgG antibodies from combinatorial libraries expressed in the cytoplasm of redox-engineered bacteria. **a** Schematic of Tat-dependent genetic selection for antigen-binding activity of full-length IgGs. In the absence of a cognate binding protein, a chimeric antigen comprised of a peptide or protein antigen of interest (orange) fused to the C-terminus of spTorA-CAT (blue) is exported out of the cytoplasm by the TatABC translocase. By localizing the chimeric antigen into the periplasm, the CAT domain is no longer able to inactivate the antibiotic chloramphenicol (Cm) by acetylation using acetyl-coenzyme A (CoA) and the host cells are rendered sensitive to antibiotic. When a cyclonal is functionally expressed in the cytoplasm of a redox-engineered *E. coli* strain (e.g., SHuffle T7 Express), it binds specifically to the chimeric antigen, thereby sequestering the CAT domain in the cytoplasm where it can efficiently detoxify Cm (green star) and conferring an antibiotic-resistant phenotype. Individual clones from the selection plate are selected, genetically identified, and functionally characterized. Schematic created with BioRender.com. **b** Schematic of pBAD24-based vector for expression of chimeric antigen constructs (left) and pCD1-based vector for expression of cyclonal IgGs (right). RBS ribosome-binding site. sp TorA signal peptide, L flexible GTSAAAG linker, Ag antigen, V_H_ variable heavy, V_L_ variable light, CH constant heavy, CL constant light.

The principle of our selection scheme for detecting antigen-binding activity in the cytoplasm of *E. coli* is illustrated in Fig. 1a. It involves the creation of a chimeric antigen biosensor in which the peptide or protein target of an antibody is genetically fused to the C-terminus of the CAT antibiotic resistance protein, which itself is modified at its N-terminus with the signal peptide derived from *E. coli* trimethylamine *N-*oxide reductase (spTorA) that is able to deliver completely folded proteins into the periplasmic compartment via the Tat export pathway^21,22^. The rationale for the design came from previous studies demonstrating the exportability of CAT when fused to Tat signal peptides^23^ as well as the use of CAT as a reliable genetic reporter of Tat export^24^.

Following expression and folding in the cytoplasm, and in the absence of a cognate binding protein, the activity of the biosensor is decreased because the CAT domain of the chimeric antigen is translocated into the periplasm where it is no longer able to inactivate the antibiotic chloramphenicol by acetylation using acetyl-coenzyme A (CoA). However, if a cyclonal that binds to the peptide or protein antigen is co-expressed, it will specifically capture the chimeric antigen and sequester CAT in the cytoplasm, leading to an increase in biosensor activity due to CAT-mediated detoxification of chloramphenicol. Because the Tat system is capable of exporting multimeric protein complexes that have assembled in the cytoplasm prior to export^25–27^, the rationale for this design is that the expected size and three-dimensional bulkiness of a cyclonal-chimeric antigen complex would exceed the capacity of the Tat system and thus be blocked for export. Indeed, the largest known natural *E. coli* Tat substrates is represented by the PaoABC heterotrimer (MW = ~135-kDa, radius of gyration, *R*_g_ = 35 Å, and maximal dimension, *D*_max_ = 120 Å^28^), whereas for a human IgG1 antibody alone the size is significantly larger (150 kDa, *R*_g_ = 53 Å, *D*_max_ = 160 Å^29^). An advantage of this approach is its simplicity as antimicrobial resistance can easily be determined in spot titer experiments, which enable the effects of mutations on protein properties (e.g., expression level, folding and assembly, binding affinity) to be phenotypically compared and quantified. Moreover, if the selectable marker is efficient enough, it should be possible to custom tailor cyclonals by selecting for variants with improved properties.

### Chimeric antigens targeted to Tat pathway inhibit cell viability

**Fig. 2:**
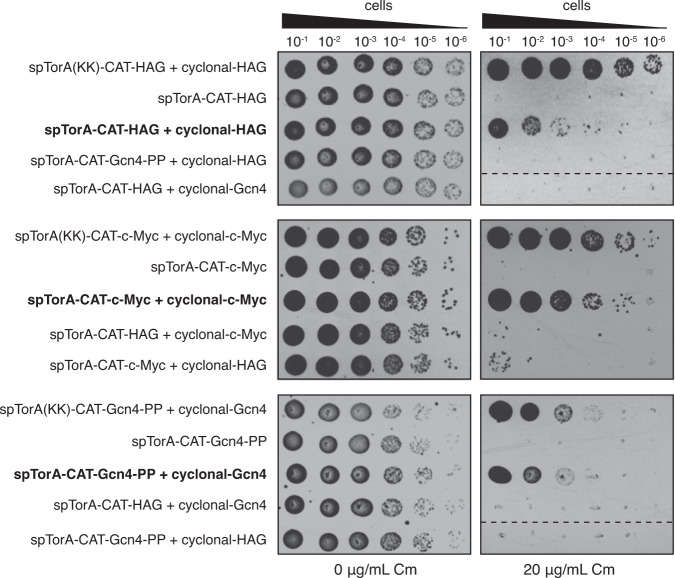
Genetic selection for cyclonal antigen-binding activity. Selective spot plating of SHuffle T7 Express cells carrying a plasmid encoding one of the chimeric antigens (spTorA-CAT-Ag or an export-defective variant spTorA(KK)-CAT-Ag) alone or with a second plasmid encoding a full-length cyclonal IgG specific for HAG, Gcn4-PP, or c-Myc antigens as indicated at left. A total of 5 μl of 10-fold serial diluted cells was plated on LB-agar supplemented with 0 or 20 μg/mL chloramphenicol (Cm) as well as 0.4% arabinose and 1 mM isopropyl β-D-thiogalactopyranoside (IPTG) to induce chimeric antigen and cyclonal expression, respectively. Cross-pairing the anti-HAG cyclonal with non-cognate c-Myc or Gcn4-PP antigens and the anti-Gcn4 cyclonal with non-cognate HAG antigen served as negative controls. Spot plating results are representative of at least three biological replicates. Dashed lines indicate merged images from discontinuous region of spot plate. Source data are provided as a Source Data file.

To validate our selection scheme, we first determined whether Tat export of these chimeric antigens yielded the expected chloramphenicol-sensitive phenotype. Specifically, we constructed a vector encoding a tripartite spTorA-CAT-Ag fusion where Ag corresponds to one of three different peptide antigens: (i) a 6-residue epitope from the hemagglutinin protein of influenza virus (HAG)^30^; (ii) a 10-residue epitope from the human c-Myc proto-oncogene product^31^; and (iii) the 47-residue basic-region leucine zipper domain of yeast Gcn4 carrying two helix-breaking proline mutations that disrupt the helical structure of the zipper and prevent its coiled-coil-mediated homodimerization (Gcn4-PP) (Fig. 1b)^27,32^. Each of these epitopes was genetically fused to the C-terminus of CAT via a seven-residue flexible linker (Gly-Thr-Ser-Ala-Ala-Ala-Gly). Importantly, all three chimeric fusions were unable to confer resistance to cells that were spot plated on agar supplemented with chloramphenicol (Fig. 2), as we had predicted. Spot plating of the same cells on agar that lacked chloramphenicol resulted in strong growth, indicating that the inability of these constructs to confer resistance on chloramphenicol was not due to a general growth defect. To determine whether chloramphenicol sensitivity was dependent on functional Tat export of the chimeric antigens, we generated mutant versions of each construct in which the two essential arginine residues of the twin-arginine motif in the spTorA signal peptide were mutated to lysines, a substitution that is well known to completely abolish export out of the cytoplasm^21,33^. Indeed, all spTorA(KK)-CAT-Ag chimeras were blocked for export as evidenced by the strong resistance to chloramphenicol that each of these constructs conferred to bacterial cells (Fig. 2). Importantly, these results indicate that the subcellular location of chimeric antigens can be discriminated by selective plating on chloramphenicol, and that cell survival depended on disrupting the Tat-dependent export of the CAT-containing chimera.

### Cyclonal expression rescues cell growth in an antigen-specific manner

To determine whether antimicrobial resistance could be positively linked to the antigen-binding activity of full-length IgGs, cyclonals specific for the HAG, c-Myc and Gcn4-PP epitopes were co-expressed with their cognate chimeric antigens. Specifically, genetically engineered SHuffle T7 Express cells, which facilitate efficient cytoplasmic disulfide bond formation^20^, were co-transformed with a plasmid encoding the cyclonal synthetic heavy and light chains, each lacking canonical export signals (Fig. 1b), along with a plasmid encoding the cognate chimeric antigen. When these cells were spot plated on agar supplemented with chloramphenicol, a clear increase in resistance was observed that was on par with the resistance conferred by the spTorA(KK)-CAT-Ag constructs expressed alone (Fig. 2). To determine whether this resistance phenotype was dependent on specific recognition of the epitope, each of the chimeric antigens was co-expressed with a non-cognate cyclonal (e.g., anti-HAG cyclonal cross-paired with spTorA-CAT-c-Myc). In all cases, there was little to no observable resistance for any of the control combinations tested, indicating that the observed antibiotic resistance was governed by antigen specificity. As above, cells grown in the absence of chloramphenicol grew robustly, indicating that cytoplasmic co-expression of these constructs had no apparent effect on cell viability. Taken together, these results unequivocally demonstrate that cyclonal IgGs sequester only their cognate chimeric antigens in the cytoplasm and significantly increase resistance by protecting cells from chloramphenicol toxicity.

**Fig. 3:**
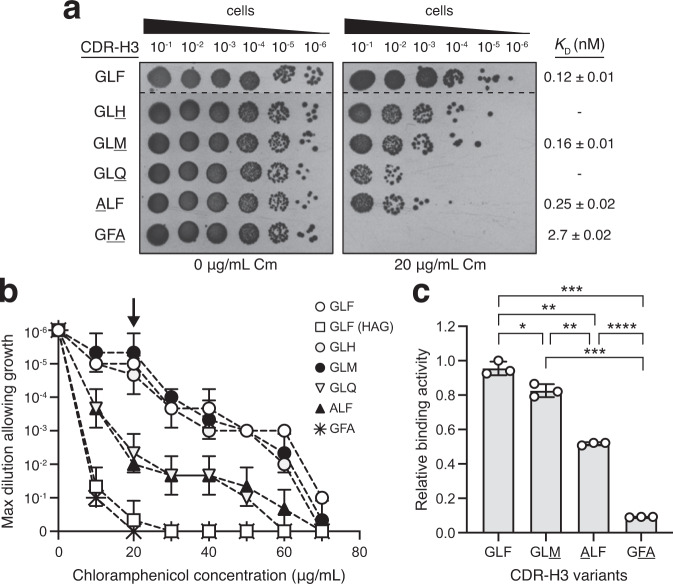
Phenotypic selection of cyclonal variants with differential antigen-binding activity. **a** Representative selective spot plating of SHuffle T7 Express cells carrying a plasmid encoding spTorA-CAT-Gcn4-PP and a second plasmid encoding anti-Gcn4 cyclonal parent (GLF) or variant with CDR-H3 mutation as indicated at left. A total of 5 μl of 10-fold serial diluted cells was plated on LB-agar supplemented with 0 or 20 μg/mL chloramphenicol (Cm) as well as 0.4 % arabinose and 1 mM IPTG to induce protein expression. Spot plating results are representative of at least three biological replicates. Dashed lines indicate merged images from two different spot plates. Binding affinity was determined for GLF, GLM, ALF, and GFA (see Supplementary Fig. 1) and values for *K*_D_ are indicated at right. **b** Survival curves for serially diluted SHuffle T7 Express cells co-expressing an anti-Gcn4 cyclonal variant along with the spTorA-CAT-Gcn4-PP reporter. Cells expressing the parental GLF cyclonal along with the non-cognate spTorA-CAT-HAG chimeric antigen served as a negative control. Overnight cultures were serially diluted in liquid LB and plated on LB-agar supplemented with Cm. Maximal cell dilution that allowed growth is plotted versus Cm concentration. Arrow in (**b**) indicates data depicted in image panel (**a**) and corresponds to 20 μg/mL Cm. Data points are the average of six biological replicates ± SD. **c** Antigen-binding activity of GLF, GLM, ALF and GFA cyclonals as determined by ELISA with purified GST-Gcn4-PP as immobilized antigen. Absorbance was measured at 450 nm and values were normalized to the maximum value obtained for GLF. Data are the average of three biological replicates ± SD. Statistical significance was determined by two-tailed *t* test with Welch’s correction (*p < 0.05; **p < 0.01, ***p < 0.001; ****p < 0.0001). Actual *p* values in from top-to-bottom and left-to-right: *p* = 0.0007, *p* = 0.0018, *p* = 0.0148, *p* = 0.0034, *p* < 0.0001, *p* = 0.0009. Source data are provided as a Source Data file.

We also tested whether the genetic selection presented here could discriminate the binding activity of different cyclonal variants. For this experiment, we focused on the anti-Gcn4 cyclonal because previous studies identified several mutations within the 5-residue heavy-chain CDR3 (CDR-H3) of single-chain Fv intrabodies whose binding activity was quantified in vivo and in vitro^27,32^. Starting with the parental CDR-H3 sequence (GLFDY, hereafter GLF), we constructed several single point mutants (GLH, GLM, GLQ, and ALF) with activity on par with or measurably lower than GLF. We also generated a double mutant (GFA) known to have greatly diminished binding activity. When cells expressing these constructs were spot plated under selective conditions, the relative resistance conferred by the five mutants was observed in the following order (from highest to lowest): GLF ≈ GLH ≈ GLM > GLQ ≈ ALF » GFA (Fig. 3a, b). These results were in harmony with the binding activities reported previously for these variants^27,32^ and with the binding affinity that we measured for several of the variants (Fig. 3c and Supplementary Fig. 1). Taken together, these results confirmed that our genetic assay was capable of distinguishing clones based on their relative affinity for antigen.

### Selection of affinity matured cyclonal variants from combinatorial libraries

Encouraged by these results, we next tested whether our selection strategy could be exploited to directly isolate Gcn4-PP binders by screening a combinatorial library of cyclonal variants. Specifically, we sought to enhance the affinity of the weakest binding cyclonal variant GFA, which had a measured equilibrium dissociation constant (*K*_D_), of 2.7 nM (Supplementary Fig. 1). For this proof-of-concept affinity maturation experiment, we chose to randomize the heavy-chain CDR3 since this variable heavy (V_H_) region is crucial for determining the specificity for most antibodies^34^. Indeed, CDR-H3 contributes important contacts to the antigen as seen in the crystal structure of the anti-Gcn4 scFv in complex with a Gcn4-derived peptide^32^. Using the GFA cyclonal variant as scaffold, we constructed a library in which the first three residues of CDR-H3 in this cyclonal were randomized using degenerate codon mutagenesis while the last two (DY) were held constant. Noting that CDR-H3 sequences frequently vary in length, we also constructed a second library based on GFA but with four fully randomized positions within a six-residue heavy-chain CDR3 sequence. The last two residues (DY) were again kept constant.

SHuffle T7 Express cells carrying the plasmid encoding spTorA-CAT-Gcn4-PP were transformed with the cyclonal libraries, after which a total of ~3 × 10^7^ clones from each library were selected on agar plates supplemented with 20 μg/mL chloramphenicol. As a negative control, SHuffle T7 Express cells carrying the plasmid encoding spTorA-CAT-Gcn4-PP along with a plasmid encoding the GFA cyclonal variant were plated similarly. After three days, >1500 colonies appeared on the library plates, while no colonies were observed on the control plates. A total of 20 positive hits were randomly chosen from plates corresponding to each library, and plasmids from all 40 were isolated and retransformed into the same reporter strain to confirm antigen-dependent resistance phenotypes. This test showed that 34 out of 40 (85%) of the originally isolated clones conferred a growth advantage to freshly transformed SHuffle T7 Express cells carrying the chimeric antigen plasmid. Sequencing of the heavy-chain CDR3 region of these positive clones revealed that most isolated sequence motifs were GLF, GLH, GLM, which all had been identified as positive binding motifs in earlier studies^27,32^. In addition, three unique motifs were also isolated: GIN, GTK, SLF from the three-residue library and GLLD from the four-residue library. Whereas GIN and GLLD were similar to the GIM and GLL motifs that we identified previously^27^, GTK and SLF were notably different. That is, all 14 unique CDR-H3 sequences reported to date contain only G or A in the first position (with a strong preference for G) and L/V/I in the second position (with a strong preference for L). While there is much weaker conservation in the third position, K has not been observed.

**Fig. 4:**
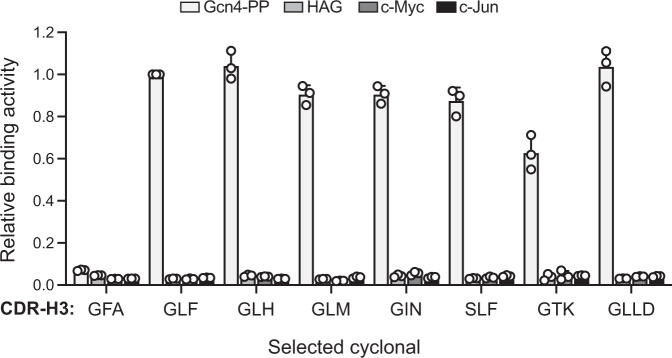
Binding analysis of affinity matured cyclonal antibodies. ELISA analysis of library-selected cyclonal antibodies derived from genetic selection with Gcn4-PP as target antigen. Binding activity and specificity of clones were evaluated by ELISA using purified GST-Gcn4-PP, GST-HAG, GST-c-Myc, or GST-c-Jun as immobilized antigen. Data were obtained for the starting GFA cyclonal antibody (*K*_D_ = 2.7 nM) and for the CDR-H3 variants that were isolated by genetic selection, namely GLF, GLH, GLM, GIN, SLF, GTK, and GLLD. It should be noted that GLF, GLH, and GLM were the most abundantly selected clones from the three-residue library. Absorbance was measured at 450 nm and values for each CDR-H3 variant were normalized to the maximum value obtained for GLF. Data are the average of three biological replicates ± SD. Source data are provided as a Source Data file.

To investigate the binding affinity and specificity of these CDR-H3s, enzyme-linked immunosorbent assay (ELISA) experiments were carried out with purified versions of Gcn4-PP, HAG, and c-Myc antigens as well as the leucine zipper of the c-Jun proto-oncogene product, which is structurally related to the Gcn4 leucine zipper but not recognized by any anti-Gcn4 scFv intrabodies^32^. Importantly, all tested cyclonals were highly specific for the cognate Gcn4-PP antigen as evidenced by the lack of measurable binding to any of the other non-cognate antigens (Fig. 4), indicating that a functional selection for antigen binding in the cytoplasm of *E. coli* has indeed occurred. It is noteworthy that the two most abundantly isolated clones, namely GLF and GLM, exhibited *K*_D_ values of 0.12 and 0.16 nM, respectively (Supplementary Fig. 1), indicating that our genetic selection strategy had successfully uncovered clones with binding affinities that were 17- and 22-fold stronger, respectively, than the binding affinity of the parental GFA clone.

### Selection of bespoke IgGs from a library of human naïve antibodies

To further demonstrate the utility of our genetic selection, we attempted to identify lead antibody candidates from a library based on a human naïve antibody repertoire. To this end, we constructed a fully human cyclonal library comprised of V_H_ and variable light (V_L_) sequences derived from transgenic mice genetically engineered with humanized immunoglobulin loci that encode a complete functional human antibody repertoire (full gamma heavy chain loci: 50 + V, 26D, 6J segments; full kappa light chain loci: 22 V, 5J segments). This library was constructed by harvesting spleen tissue from humanized mice and preparing RNA from the recovered spleens. Next, splenic RNA was subjected to RT-PCR to generate cDNA libraries encoding V_H_ and V_L_ chain domain sequences. From the RNA-derived cDNA libraries, V_H_ and V_L_ genes were PCR amplified and ligated in-frame to a human IgG framework in plasmid pCOLADuet^TM^−1, resulting in a fully human, naïve cyclonal library.

**Fig. 5:**
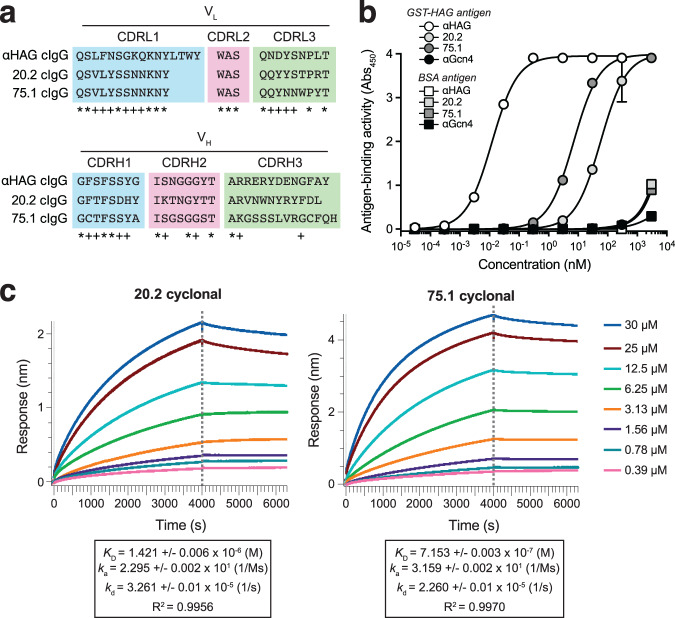
Binding analysis of lead cyclonal antibodies selected from combinatorial libraries. **a** Comparison of CDRs for murine anti-HAG cyclonal and human 20.1 and 75.1 cyclonals. Asterisk (*) indicates residue shared by all three clones; plus sign (+) indicates residue shared by two clones. **b** ELISA analysis of library-selected cyclonal antibodies derived from genetic selection with HAG as target antigen. Binding activity and specificity of the lead human cyclonal antibodies, 20.2 and 75.1, as well as the murine anti-HAG and anti-Gcn4 cyclonals were evaluated by ELISA using purified GST-HAG or BSA as immobilized antigen. Absorbance was measured at 450 nm for each cyclonal and the resulting measurements were analyzed using GraphPad Prism 9 to determine binding affinity. Data are the average of three biological replicates ± SD. **c** Biolayer interferometry (BLI) kinetic assays were performed to measure kinetic binding constants (*k*_a_, *k*_d_) and equilibrium binding constants (*K*_D_) for the interaction between the 20.2 and 75.1 cyclonals and HAG peptide. Biotinylated GST-HAG was immobilized on streptavidin (SA)-coated sensors and subsequently used to bind protein A-purified cyclonals in solution. Response data are representative of replicate BLI experiments (*n* = 2). Source data are provided as a Source Data file.

To select IgGs specific for the HAG antigen, SHuffle T7 Express cells carrying the plasmid encoding spTorA-CAT-HAG were transformed with the newly created cyclonal library, after which a total of ~2 × 10^6^ clones from the library were selected on agar plates supplemented with a range of chloramphenicol concentrations. A total of 18 positive hits were randomly chosen from plates supplemented with a range (10–75 μg/mL) of chloramphenicol, with no colonies appearing on control plates over this same range of chloramphenicol. Plasmids from all 18 were isolated and retransformed into the same reporter strain to confirm antigen-dependent resistance phenotypes. This test showed that 14 out of 18 (78%) of the originally isolated clones conferred a growth advantage to freshly transformed SHuffle T7 Express cells carrying the chimeric antigen plasmid (Supplementary Fig. 2). Sequencing of the V_H_ and V_L_ domains identified two unique hits, 20.2 and 75.1, that were each isolated multiple times (2× and 3×, respectively) and thus chosen for further analysis. A comparison of their CDRs revealed that, despite their uniqueness, clones 20.2 and 75.1 had identical CDR-L1 and CDR-L2 sequences and multiple residues in common in all other CDRs except for CDR-H3, which exhibited the greatest sequence divergence (Fig. 5a). IgBlast analysis^35^ of these sequences confirmed their human origin and revealed putative germline sequences from which each may have arisen (Supplementary Fig. 3). Interestingly, clones 20.2 and 75.1 also shared a few common CDR residues with the unrelated murine anti-HAG IgG that served as the positive control (Fig. 5a), suggesting the existence of conserved residues that may be important for antigen binding.

To investigate the binding affinity and specificity of these hits, the 20.2 and 75.1 cyclonals were affinity purified and subjected to antigen-binding analysis by ELISA. Importantly, both cyclonals were observed to bind strongly to the immobilized HAG antigen but exhibited little detectable binding to the non-cognate antigen bovine serum albumin (BSA) that served as a negative control (Fig. 5b), confirming that these genetically selected hits were specific for their intended target. The dissociation constants for 20.2 and 75.1 were estimated to be 1.42 and 0.72 μM, respectively (Fig. 5c), which represent reasonably strong affinities following just a single round of selection from a non-immune library that was generated from the naturally present diversity of the circulating B cell repertoire in a humanized mouse model. By way of comparison, the anti-HAG cIgG exhibited significantly stronger binding affinity with an estimated dissociation constant of 0.75 nM (Supplementary Fig. 4). However, this stronger affinity was expected given that the variable regions of this antibody were derived from an IgG that was isolated by hyperimmunizing mice with a synthetic HAG peptide coupled to the strongly immunogenic keyhole limpet hemocyanin (KLH) carrier protein^36^. Taken together, these results confirm the ability of our genetic selection to identify lead antibody candidates from naïve human antibody libraries.

## Discussion

In this study, we describe a genetic selection for rapid and reliable isolation of full-length IgG antibodies from combinatorial libraries expressed in the cytoplasm of *E. coli*. This selection strategy is significant given the outsized impact that recombinant antibodies have made on biomedical research, and increasingly on molecular medicine. Indeed, straightforward technologies that aid in the discovery of mAbs for clinical and therapeutic development remain in high demand. To this end, we designed and validated a high-throughput assay that effectively links the binding activity of recombinantly expressed IgG antibodies called cyclonals with antibiotic resistance conferred by capture of engineered chimeric antigen biosensors. Using a set of cyclonal variants with the same specificity for one epitope, the leucine zipper domain of yeast Gcn4, we showed that this assay can discriminate antigen-specific cyclonals based on their relative affinities. That is, cells carrying plasmids encoding specific antigen-antibody pairs exhibited an observable fitness advantage over cells carrying plasmids encoding non-specific pairs.

The utility of this approach was subsequently revealed by two combinatorial library-based enrichment experiments. The first experiment involved affinity maturation of an existing anti-GCN4 cyclonal antibody by selection of high-affinity variants from a library that was generated by randomizing the CDR-H3 sequences of the parental cyclonal. The second experiment involved discovery of additional anti-GCN4 cyclonals by selection of lead antibodies from a large, naïve library of IgG antibody sequences comprised of human V_H_ and V_L_ variable region genes obtained from a humanized mouse. These proof-of-concept library selections revealed the potential of our genetic assay for both identification of bespoke antigen-specific IgG antibody leads and affinity maturation of existing IgG antibodies. Regarding the latter, we anticipate that further affinity maturation of antibodies could be achieved through multiple rounds of mutagenesis and selection in which the chloramphenicol concentration is incrementally increased in each round. Although not directly demonstrated here, we note that similar toggling of antibiotic concentration was used by our group to mature the solubility of single-chain antibodies using a survival-based selection for intracellular protein folding in *E. coli*^37^.

Importantly, the results presented here provide proof-of-concept demonstration of bacterial genetic selection applied to the discovery of full‐length IgG antibodies. Genetic selections are attractive as they link a desired property, in this case antigen-binding activity, to the fitness of the host organism. To date, a handful of genetic selections have been reported for isolating functional antibodies in bacteria and yeast; however, these have only been demonstrated for scFvs and other small formats^25,27,37–43^ but not full-length IgGs. Indeed, the vast majority of recombinant antibody screening platforms in microorganisms make use of scFv or Fab antibodies^8^. While these formats are relatively easy to produce in bacteria and yeast, they are monovalent proteins that typically lack avidity effects which can be important for reducing antigen off-rates and for enhancing the recovery of low‐affinity binders^44^. Moreover, these monovalent formats are generally unsuitable for therapeutic development and must be converted to full-length IgGs prior to use in the clinic. Unfortunately, the conversion process requires additional cloning steps and can result in loss of binding activity^12^. By leveraging full-length IgG expression in the bacterial cytoplasm^19^, our approach obviates the need for post-selection molecular reformatting.

In the context of full-length IgG antibodies, cell surface display has been the predominant technology for screening recombinantly expressed libraries^10–16^. An advantage of cell surface display methods, especially those involving eukaryotic cells, is the intrinsic ability of these hosts to introduce important post-translational modifications (e.g., disulfide bonds, *N*-linked glycosylation) via processes that are integrated with the secretory pathway. In the context of disulfide bonds, we circumvented the need for translocation to a more oxidizing compartment by using *E. coli* SHuffle cells, which have been engineered with a strongly oxidizing cytoplasmic compartment that favors disulfide bond formation including in IgG antibodies^19^. This is significant because trafficking across biological membranes can complicate expression/selection and can introduce biases due to the elimination of IgG clones that transit the secretory pathway inefficiently or not at all. As for *N*-glycosylation, which plays a crucial role in certain Fc-mediated effector functions such as antibody-dependent cell-mediated cytotoxicity (ADCC), this is an inherent limitation of our bacterial selection technology compared to display technologies using mammalian cells or glycoengineered yeast that can install human-like *N*-glycan structures. However, because Fc glycans are not required for antigen-binding activity, bacterial selection should be ideally suited for lead antibody discovery or antibody affinity maturation, as we demonstrated here. Following antibody discovery and/or optimization in *E. coli*, one would likely need to switch to a different host for the development of antibodies that require Fc glycans. Alternatively, one could introduce mutations to the Fc domain that restore Fc-mediated effector functions in the absence of *N-*glycosylation^45,46^ and are compatible with cytoplasmic expression in SHuffle cells^19^. Despite the extra effort involved in switching hosts or generating additional mutations, we feel this is more than offset by the convenience and time-savings afforded by our greatly simplified, agar plate-based genetic selection method for interrogating libraries and isolating clones.

A notable caveat to our method is that target antigens must be solubly expressed in the cytoplasm of SHuffle cells to be compatible with our genetic selection. It is fortuitous therefore that SHuffle cells can reportedly express a wide range of recombinant proteins, including those that require disulfide bonds for proper folding, as soluble products in the cytoplasm^20^. Another caveat worth mentioning is that antigens exceeding a certain size could yield CAT-antigen fusions that exceed the threshold of the Tat translocase and thus would be incompatible with our selection strategy. While no definitive upper limit has been determined for the size of a molecule that can be exported by the Tat translocase, the largest naturally occurring Tat substrate identified to date is *E. coli* PaoABC (MW = ~135-kDa, radius of gyration, *R*_g_ = 35 Å, and maximal dimension, *D*_max_ = 120 Å^28^), which is reliably translocated to the periplasm in *E. coli* cells^25^. Based on this information, we suspect that proteins significantly larger than ~135-kDa will be export incompetent. Indeed, the data presented here shows that IgG binding to a CAT-Ag fusion results in the formation of a multimeric complex that is too large to be exported by the Tat translocase. Hence, to be compatible with our assay, we anticipate that antigens will need to be in the 50–60 kDa range or smaller.

Another advantage of our approach is that genetic selection is intrinsically high throughput, enabling cyclonal variants with desirable binding activity to be readily isolated from large libraries by simple transformation and plating of bacteria without needing to purify, label, or immobilize the target antigen. While not directly demonstrated here, our selection strategy should permit selection of very large libraries (>10^11^). Screens, on the other hand, require every member of a library to be analyzed, making the process of identifying clones with beneficial mutations much more labor intensive. For example, many previous display-based methods involve fluorescence activated cell sorting (FACS), a very powerful high‐throughput screening methodology; however, interrogating a library of >10^8^ cells using FACS is dependent on expensive instrumentation, time‐consuming, and technically challenging^12,17^. In fact, for combinatorial libraries of this size, an initial phage display screening process was required to reduce the initial library to a size that was manageable by FACS^12^.

A final advantage is that unlike nearly all other full-length IgG screening methods that require tethering of the antibody to a cellular membrane, using either fusion to a membrane anchoring polypeptide^15,16^ or introduction of a secretion-and-capture step prior to antigen binding^10–14^, our ‘membrane-less’ approach does not depend on physical display of the antibody. In fact, our method of cytoplasmic IgG expression circumvents membrane translocation of these large macromolecules altogether, which is important because traversing tightly sealed biological membranes is a rate limiting and energy intensive step that can serve as a potential source of selection bias in these previous IgG screening methods. While other membrane-less IgG screening strategies exist, in particular methods for encapsulating single IgG antibody secreting cells in water-in-oil droplets^47,48^ or gel microdroplets^49–52^, construction of such drop-based secretor cell libraries is non-trivial, often involving microfluidics, and screening must typically be performed in conjunction with FACS, which introduces additional challenges as discussed above. It should also be pointed out that because our selection requires no modification of the IgG, the selected plasmid can be used directly for functional IgG expression without any subcloning, thereby streamlining the process from selection to expression of IgG antibody candidates.

In conclusion, we have demonstrated a promising methodology for stringent selection of full-length IgG antibodies from combinatorial libraries with the potential to yield high-affinity binders with selective target binding characteristics. In the future, we anticipate that this system will find use in the isolation of bespoke antibodies by functionally interrogating more complex libraries comprised of naïve antibody repertoires as well as in the engineering of ultra-high affinity IgG antibodies by affinity maturing parental antibody sequences using directed evolution workflows. With these and other imagined uses, our recombinant antibody selection technology represents a highly complementary addition to the antibody engineering toolkit that should facilitate discovery of antibody-based research reagents, diagnostics, and biopharmaceuticals in the years to come.

## Methods

### Bacterial strains

*E. coli* strain DH5α was used for plasmid construction while SHuffle T7 Express (New England Biolabs)^20^ was used for cyclonal expression and library selections. Protein antigens for immunoassays including GST-Gcn4-PP, GST-HAG, GST-c-Myc, and GST-c-Jun were expressed using *E. coli* T7 Express (New England Biolabs).

### Plasmid construction

The pBAD24 plasmid^53^ was used for construction of all spTorA-CAT-Ag chimeric antigen reporter fusions. First, a PCR product corresponding to spTorA-JunLZ-FLAG^27^, encoding the signal peptide of *E. coli* TorA (spTorA) fused to the N-terminus of the c-Jun leucine zipper (JunLZ) was cloned between the NheI and HindIII restriction sites of pBAD24, yielding plasmid pBAD24-spTorA-JunLZ-FLAG. Next, the gene encoding CAT was PCR-amplified from pACYC-Duet^TM^−1 (Novagen) to include a 3’ flexible linker (GTSAAAG) flanked by SalI and SpeI restriction sites. At the same time, the gene encoding Gcn4(7P14P)^27,32^, encoding a double proline mutant of the leucine zipper domain of Gcn4 that reduces its propensity for homodimerization, was PCR-amplified from pBAD33-Gcn4(7P14P)-Bla^27^ to include the same flexible linker sequence at the 5’ end. The two resulting PCR products were fused by overlap extension PCR and the overlap product was cloned between the XbaI and HindIII sites of pBAD24-spTorA-JunLZ-FLAG, yielding plasmid pBAD24-spTorA-CAT-Gcn4-PP. Genes encoding the HAG (DVPDYA) and c-Myc (EQKLISEEDL) epitopes were constructed by annealing complementary oligonucleotides, and were subsequently cloned in place of Gcn4(7P14P) between SpeI and HindIII sites in pBAD24-spTorA-CAT-Gcn4-PP, yielding plasmids pBAD24-spTorA-CAT-HAG and pBAD24-spTorA-CAT-c-Myc.

The creation of all bacterial IgG expression constructs involved plasmid pCOLADuet^TM^−1 (Novagen), which is designed for the co-expression of two target genes from independent upstream T7 promoter/lac operator regions. First, the light chain genes (V_L_-mC_L_κ) for anti-HAG and anti-Gcn4 were PCR-amplified from pMAZ360-cIgG-HAG and pMAZ360-cIgG-Gcn4^19^, respectively, and cloned between NcoI and NotI sites of pCOLA-Duet^TM^−1, yielding plasmids pCD1-cLC-HAG and pCD1-cLC-Gcn4, respectively. Next, the heavy chain Fab genes (V_H_-mC_H_1) were PCR-amplified from the same pMAZ360 templates and cloned between NdeI and AscI sites in pCD1-cLC-HAG and pCD1-cLC-Gcn4, yielding plasmids pCD1-cFab-HAG and pCD1-cFab-Gcn4. Finally, the heavy chain Fc genes (hFc) were PCR-amplified from the pMAZ360 template plasmids and cloned between AscI and XhoI sites in pCD1-cFab-HAG and pCD1-cFab-Gcn4, yielding plasmids pCD1-cIgG-HAG and pCD1-cIgG-Gcn4.

To construct the anti-c-Myc cyclonal, the gene encoding the V_L_ domain of scFv-3DX^31^ was PCR-amplified using primers that introduced a sequence overlapping with the mouse constant light chain kappa domain (mC_L_κ). In parallel, the gene encoding mCLκ was PCR-amplified with primers that introduced a 5’ sequence overlapping with the V_L_ of scFv-3DX. The resulting PCR products were assembled by overlap extension PCR, generating the anti-c-Myc light chain (V_L_-mC_L_κ). Similarly, the gene encoding the V_H_ domain of scFv-3DX was PCR-amplified using primers that introduced a sequence overlapping with the mFab/hFc heavy chain constant domains. At the same time, the mFab/hFc constant heavy chain domains were amplified with primers that introduced a 5’ sequence overlapping with V_H_ of scFv-3DX. Again, the resulting products were assembled by overlap extension PCR, generating the anti-c-Myc heavy chain (V_H_-mC_H_1-hFc). The light chain and heavy chain products were then cloned between NcoI/NotI and NdeI/XhoI sites, respectively, of pCOLADuet^TM^−1, yielding the plasmid pCD1-cIgG-c-Myc. The heavy-chain CDR3 cyclonal variants GLH, GLM, GLQ, ALF, and GFA were constructed by site-directed mutagenesis of the parental GLF cyclonal heavy chain sequence. Plasmids pET28a-GST-Gcn4-PP and pET28a-HAG were described previously^27^. An identical strategy was used to construct pET28a-GST-c-Myc and pET28a-GST-c-Jun. All plasmids constructed in this study were confirmed by sequencing at the Cornell Biotechnology Resource Center.

### Selective growth assays

Chemically competent SHuffle T7 Express cells were transformed with one of the pBAD24-spTorA-CAT-Ag plasmids along with a pCD1-cyclonal plasmid, and spread on Luria-Bertani (LB)-agar plates supplemented with 25 μg/mL spectinomycin (Spec), 25 μg/mL kanamycin (Kan), and 50 μg/mL ampicillin (Amp), and cultured overnight at 37 °C. The next day, 3 mL of LB supplemented with appropriate antibiotics was inoculated with three freshly transformed colonies and incubated at 30 °C for 12–18 h. Cells carrying the pBAD24-spTorA-CAT-Ag and pCD1-cyclonal plasmids were normalized to an absorbance at 600 nm (Abs_600_) ≈ 2.5 (2.5 × 10^9^ cells/mL). Cells were then serially diluted ten-fold in liquid LB, and 5 μL of each dilution was spotted on selective induction plates supplemented with 25 μg/mL Spec, 25 μg/mL Kan, 50 μg/mL Amp, 1 M IPTG, 0.2% (w/v) arabinose, and varying concentrations of Cm. The plates were then incubated at 30 °C for 24–48 h. Image Lab 6.1 software (Bio-Rad) was used for collecting spot plate images.

### Library construction

For affinity maturation of the GFA cyclonal, random mutagenesis of the first three residues of CDR-H3 was performed using NDT and NNK degenerate codons. The resulting library encoded anti-Gcn4 cyclonals with heavy-chain CDR3 motifs of the form XXXDY, where X was encoded by either the NDT codon (encoding 12 amino acids: N, S, I, H, R, L, Y, C, F, D, G, and V; and no stop codons) or NNK (encoding all amino acids and one stop codon. Random mutagenesis of the CDR-H3 was achieved by amplifying the entire pCD1-cIgG-Gcn4(GFA) plasmid by inverse PCR with degenerate NDT and NNK primers encoding the three randomized codons within CDR-H3. The resulting linear PCR product was circularized by blunt-end ligation to produce the plasmid library. The circularized products were used to transform electrocompetent DH5α cells. The transformed cells were cultured overnight in 100 mL LB supplemented with 50 μg/ml Kan. Plasmid DNA was purified by maxiprep from the overnight culture for selection experiments. Random mutagenesis of the first four residues of CDR-H3 was performed identically using a degenerate NDT primer to generate six-residue heavy-chain CDR3 motifs of the form XXXXDY.

To construct a human naïve antibody library for identification of lead IgG candidates against the HAG epitope, we harvested RNA from the spleen of a 6-week-old, female humanized mouse using the RNeasy Midi Kit (QIAgen) according to the manufacturer’s instructions. Humanized ATX-GK mice with complete functional human gamma heavy chain and kappa light chain on a BL/6 background (MHC Haplotype H-2b) were obtained from Alloy Therapeutics. Mice were housed under the following environmental conditions to reduce stress: 14-hour light/10-hour dark cycle and temperature of ~70 F with ~50% humidity. This work was carried out under Protocol 2012-0132 approved by the Cornell University Institutional Animal Care and Use Committee (IACUC). RT-PCR was performed using the SuperScript III kit (ThermoFisher) using 500 ng RNA and random hexamers according to the manufacturer’s instructions. Immunoglobulin V_H_ and V_L_ chains were amplified from the cDNA preparations using the primers described in Supplementary Dataset 1 and were randomly cloned into the pCOLADuet^TM^−1 plasmid through Gibson Assembly using the E. cloni 10 G SUPREME electrocompetent cells (Lucigen, >4 × 10^10^ CFU/g pUC DNA). The cell library was expanded in LB supplemented with 50 μg/mL Kan, and the library DNA was purified using the Maxiprep kit (QIAgen) according to manufacturer’s instructions.

### Library selection

To perform library selections for affinity maturation, electrocompetent SHuffle T7 Express cells carrying pBAD24-spTorA-CAT-Gcn4-PP were transformed with the purified anti-Gcn4 cyclonal libraries. Transformants were incubated in SOC media at 37 °C for 1 h without antibiotics and then cultured overnight in LB supplemented with appropriate antibiotics and 0.2% glucose. The next day, overnight cells were normalized to Abs_600_ ≈ 2.5 and serially diluted to 10^−3^, 10^−4^, and 10^−5^. A total volume of 225 µL of each dilution was plated on LB-agar supplemented with 15–30 μg/mL Cm, 0.2% (w/v) arabinose, and 1 mM IPTG and cultured at 30 °C for 72 h. At the same time, cells transformed with plasmid pBAD24-spTorA-CAT-Gcn4-PP and pCD1-cIgG-Gcn4(GFA) and were treated in an identical manner as library cells and served as a negative control. Clones that appeared on selective plates were picked at random and resistance to Cm was verified by isolating plasmid DNA, retransforming SHuffle T7 Express cells, and performing selective spot plating with the freshly transformed cells. Plasmid DNA of verified positive hits was sequenced at the Cornell Biotechnology Resource Center.

Selection of clones from naïve human antibody libraries was performed similarly except that reporter plasmid pBAD24-spTorA-CAT-HAG was used to express the HAG antigen and serial dilutions of 10^−0^, 10^−1^, 10^−2^, and 10^−3^ were plated on Cm ranging in concentration from 10 to 75 μg/mL. *E. coli* cells transformed with plasmid pBAD24-spTorA-CAT-HAG and pCD1-cIgG-HAG served as a positive control while cells transformed with plasmid pBAD24-spTorA(KK)-CAT-HAG and pCD1-cIgG-HAG served as a negative control.

### Preparation of soluble cell extracts and cyclonal purification

A single colony of SHuffle T7 Express carrying one of the pCD1-cyclonal plasmids was used to inoculate 2 mL LB supplemented with appropriate antibiotics, and grown overnight at 30 °C. The next day, 5 ml of fresh LB supplemented with appropriate antibiotics was inoculated 1/100 with the overnight culture and cells were grown at 30 °C until reaching Abs_600_ ≈ 0.7. At this point, cyclonal expression was induced by addition of 0.1 mM IPTG, after which cells were incubated an additional 16 h at RT or 30 °C. Cells were harvested by centrifugation before preparation of lysates. Cells expressing recombinant proteins were harvested by centrifugation (4000 × *g*, 4 °C) and resuspended in PBS and 5 mM EDTA. Cells were lysed in an ice-water bath by sonication (Branson sonifier 450; duty cycle 30%, output control 3) using four repetitions of 30 s each. The insoluble fraction was removed by centrifugation (21,000 × *g*, 4 °C) and the supernatant was collected as the soluble fraction.

Cyclonals were purified from the soluble fraction of lysates derived from SHuffle T7 cells transformed with pCD1-cyclonal plasmids^19^. Briefly, cells were incubated in LB media supplemented with 50 μg/mL Kan at 30 °C until reaching Abs_600_ ≈ 0.6. Cyclonal expression was induced with 0.05 mM IPTG for 48 h at 16 °C. Harvested cells were resuspended in PBS with 5 mM EDTA and protease inhibitor (ThermoFisher) equal to one-tenth of the original culture volume and lysed using an EmulsiFlex-C5 homogenizer (Avestin). The cell lysate was clarified by centrifugation at 17,700 × *g* at 4 °C for 25 min. Protein A agarose resin (Mabselect SuRe) was equilibrated with PBS and then mixed with the soluble lysates. The resin-soluble lysate mixture was incubated at room temperature with end-over-end mixing for 2 h. The mixture was then applied to a polypropylene gravity column and the soluble lysate was allowed to completely pass through the column. The protein A agarose was then washed with PBS and cyclonals were eluted form the column with 0.1 M glycine-HCl (pH 3) in 1 M Tris (pH 9) at a 1:5 ratio. Purified fractions were applied to protein concentrators (50 K MWCO; ThermoFisher) to change the buffer to PBS.

### ELISA

To quantify binding activity and specificity of cyclonals, the GST-Gcn4-PP, GST-HAG, GST-c-Myc, and GST-c-Jun fusion proteins were expressed in *E. coli* T7 express cells and purified using Ni-NTA affinity resin according to standard protocols. Next, Costar 96-well ELISA plates (Corning) were coated overnight at 4 °C with 50 µL of 10 µg/mL of each of the different GST fusions, in 0.05 M sodium carbonate buffer (pH 9.6). After blocking in PBST with 3% (w/v) milk (PBSTM) for 1–3 h at room temperature, the plates were washed four times with PBS buffer and incubated with serially diluted soluble fractions of crude cell lysates for 1 h at room temperature. Cyclonal IgG-containing lysates were prepared as described above and quantified by Bradford assay. An equivalent amount of total protein (typically 8–64 mg) corresponding to each cyclonal sample was applied to the plate. After washing four times with the same buffer, 50 µL of 1:5000-diluted rabbit anti-human IgG (Fc) antibody–HRP conjugate (ThermoFisher, cat # 31423) in PBSTM was added to each well for 1 h. The 96-well plates were then washed six times with PBST. After the final wash, 200 µL SigmaFAST™ OPD solution (Sigma-Aldrich) was added and incubated in each well in the dark for 30 min. The HRP reaction was then terminated by the addition of 50 µL 2 M H_2_SO_4_ to the wells. Following reaction quenching, the absorbance of each well was measured at 450 nm. To quantify binding affinity of select cyclonals, an identical ELISA protocol was followed except that cyclonals were subjected to protein A-mediated purification as described above prior to loading onto ELISA plates. The determination of equilibrium dissociation constant, *K*_D_, was performed using GraphPad Prism 9 for MacOS (version 9.2.0) software^54^.

### Biolayer interferometry analysis

The dissociation constant for protein-A purified cyclonals was measured by biolayer interferometry (BLI) assays using an Octet RH16 instrument (Sartorious) at 30 °C with shaking at 1000 rpm. Kinetic analysis using streptavidin (SA) biosensor tip (Sartorious) was performed as follows: (1) baseline: 30 s immersion in buffer (0.5% BSA, 0.02% Tween 20 in PBS, 0.2 μm filtered); (2) loading: 60 s immersion in a solution with biotinylated-His-GST-HAG, 0.2 μg/mL; (3) baseline: 60 s immersion in buffer; (4) association: 4000 s immersion in solution with cyclonal at noted concentration; (5) dissociation: 2300 s immersion in buffer. The kinetic data was analyzed using the Octet Analysis Studio software v12.2.2.26 (Sartorious). Raw data were corrected by subtracting the signal obtained from traces performed with cIgGs with unloaded sensor tips. 1:1 binding model was used for curve fitting and kinetic constants were obtained by performing a global fit.

### Statistical analysis

Statistical significance between groups was determined by unpaired *t*-test with Welch’s correction using GraphPad Prism software for MacOS (version 9.4.1). Statistical parameters including the definitions and values of *n*, *p* values, and SDs are reported in the figures and corresponding figure legends.

### Reporting summary

Further information on research design is available in the Nature Portfolio Reporting Summary linked to this article.

## Supplementary information

Supplementary Information

Reporting Summary

Description of Additional Supplementary Files

Supplementary Dataset 1

## Data Availability

All data generated or analyzed during this study are included in this article and its Supplementary Information/Source Data file. Source data are provided with this paper.

## Source data

Source Data File
