# Supplementary material for: Isolation of full-length IgG antibodies from combinatorial libraries expressed in the cytoplasm of Escherichia coli
Source: Nat Commun. 2023 Jun 14;14:3514. doi: 10.1038/s41467-023-39178-x (PMC10267130; doi:10.1038/s41467-023-39178-x)
Supplement: Supplementary file 1 — Supplementary Information [file 41467_2023_39178_MOESM1_ESM.pdf]

Supplementary Information

Isolation of full-length IgG antibodies from combinatorial libraries expressed in the cytoplasm of *Escherichia coli*

Michael-Paul Robinson[1†], Jinjoo Jung[1†], Natalia Lopez-Barbosa[1*], Matthew Chang[1], Mingji Li[1], Thapakorn Jaroentomeechai[1], Emily C. Cox[2], Xiaolu Zheng[1], Mehmet Berkmen[2] and Matthew P. DeLisa[1,2,4*]

[1]Robert F. Smith School of Chemical and Biomolecular Engineering, Cornell University, Ithaca, NY 14853 USA
[2]Biomedical and Biological Sciences, College of Veterinary Medicine, Cornell University, Ithaca, New York 14853 USA
[3]New England Biolabs, 240 County Road, Ipswich, Massachusetts 01938, USA
[4]Cornell Institute of Biotechnology, Cornell University, Ithaca, NY 14853, USA

[†]These authors have contributed equally to this work

*Address correspondence to: Matthew P. DeLisa, Robert Frederick Smith School of Chemical and Biomolecular Engineering, Cornell University, Ithaca, NY 14853. Tel: 607-254-8560; Email: md255@cornell.edu

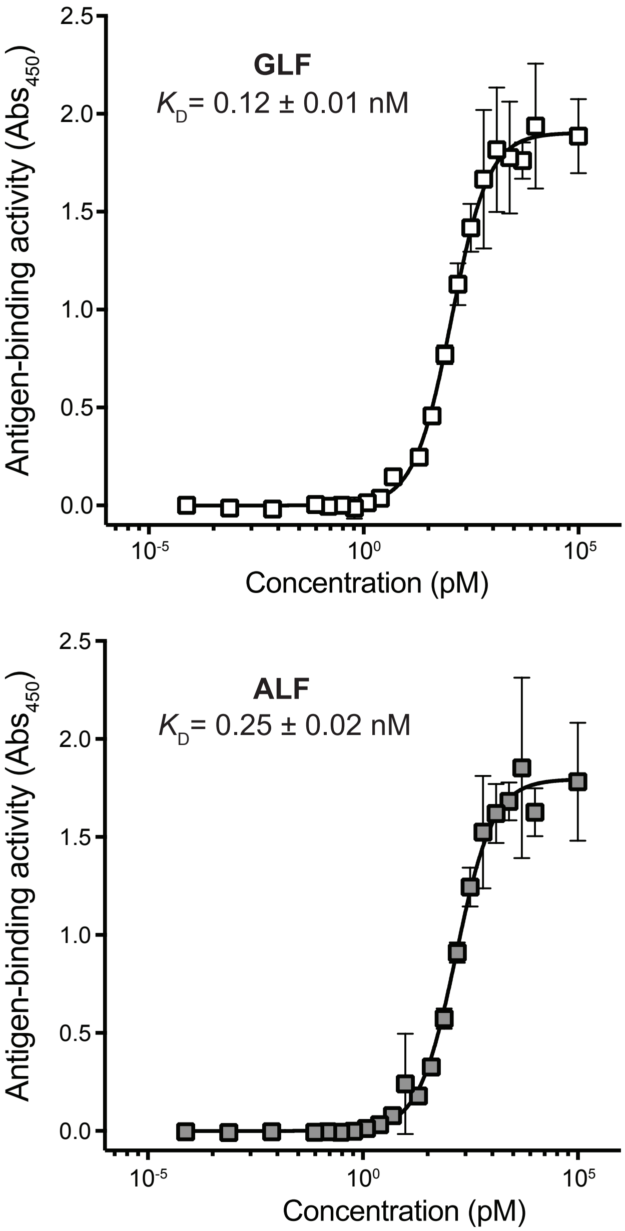

Supplementary Figure 1. Affinity determination of cyclonal antibodies. Binding affinity of cyclonals GLF, GLM, ALF, and GFA determined by ELISA with purified GST-Gcn4-PP as immobilized antigen. Absorbance was measured at 450 nm for each cyclonal variant and the resulting measurements were analyzed using GraphPad Prism 9 to determine K_D. Data are the average of biological replicates (n = 3) and error bars represent the standard deviation. Source data are provided as a Source Data file.

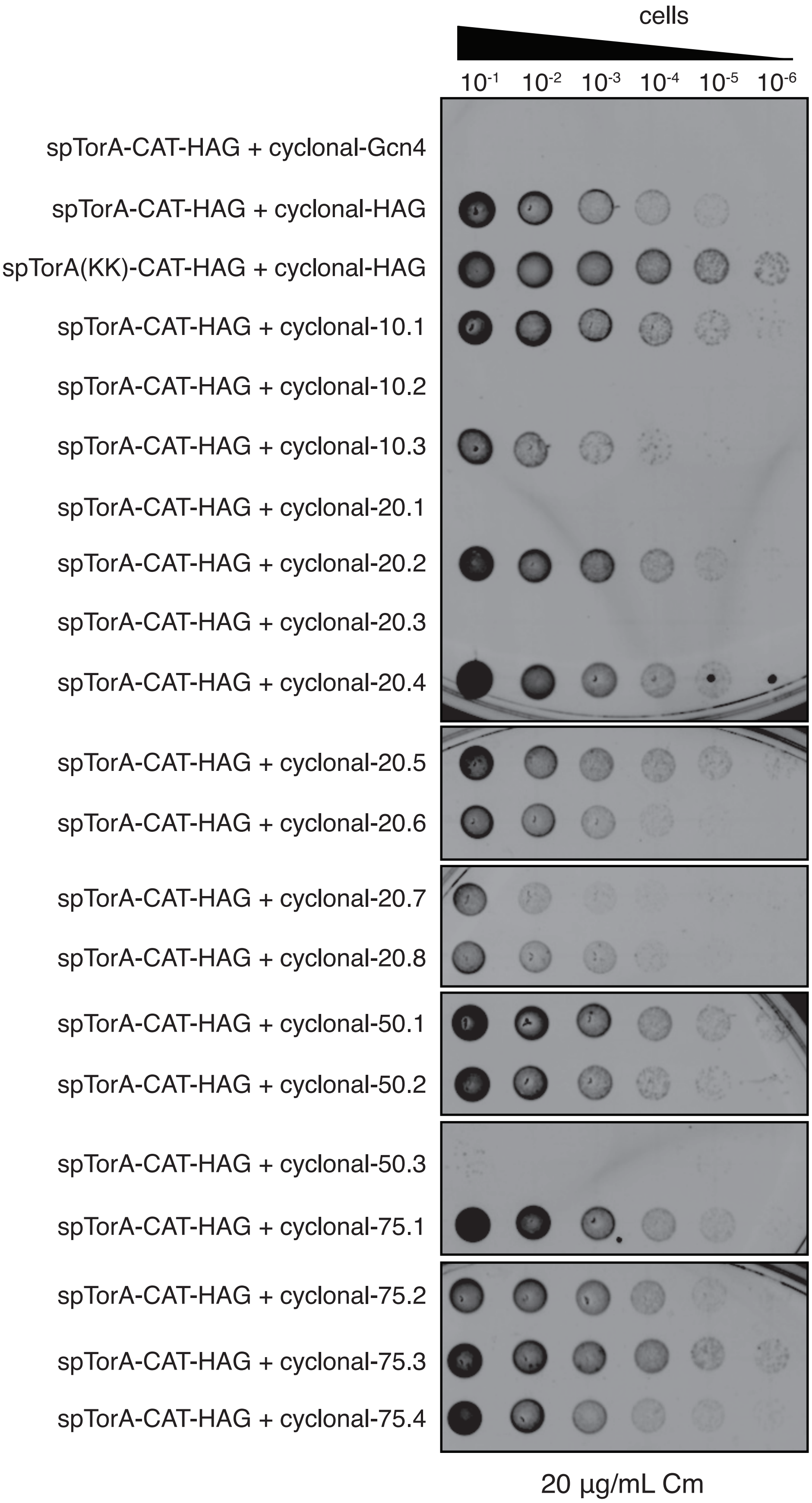

Supplementary Figure 2. Genetic selection of lead cyclonal IgG candidates against HAG peptide. Selective spot plating of SHuffle T7 Express cells carrying a plasmid encoding spTorA-CAT-HAG along with a second plasmid encoding a cyclonal IgG candidate that was selected for binding to the HAG peptide antigen. A total of 5 µl of 10-fold serial diluted cells was plated on LB-agar supplemented with 20 µg/ml chloramphenicol (Cm) as well as 0.4 % arabinose and 1 mM isopropyl β-D-thiogalactopyranoside (IPTG) to induce chimeric antigen and cyclonal expression, respectively. Cross-pairing the non-cognate Gcn4 cyclonal with HAG antigen or the HAG cyclonal with export-defective spTorA(KK)-CAT-HAG served as negative controls. Spot plating results are representative of at least three biological replicates.

a

Cyclonal	Light chain V	Light chain J	Heavy chain V	Heavy chain D	Heavy chain J
20.2	IGKV4-1*01	IGKJ1*01	IGHV3-72*01	IGHD1-7*01	IGHJ2*01
75.1	IGKV4-1*01	IGKJ1*02	IGHV3-23*04	IGHD6-13*01	IGHJ1*01

b

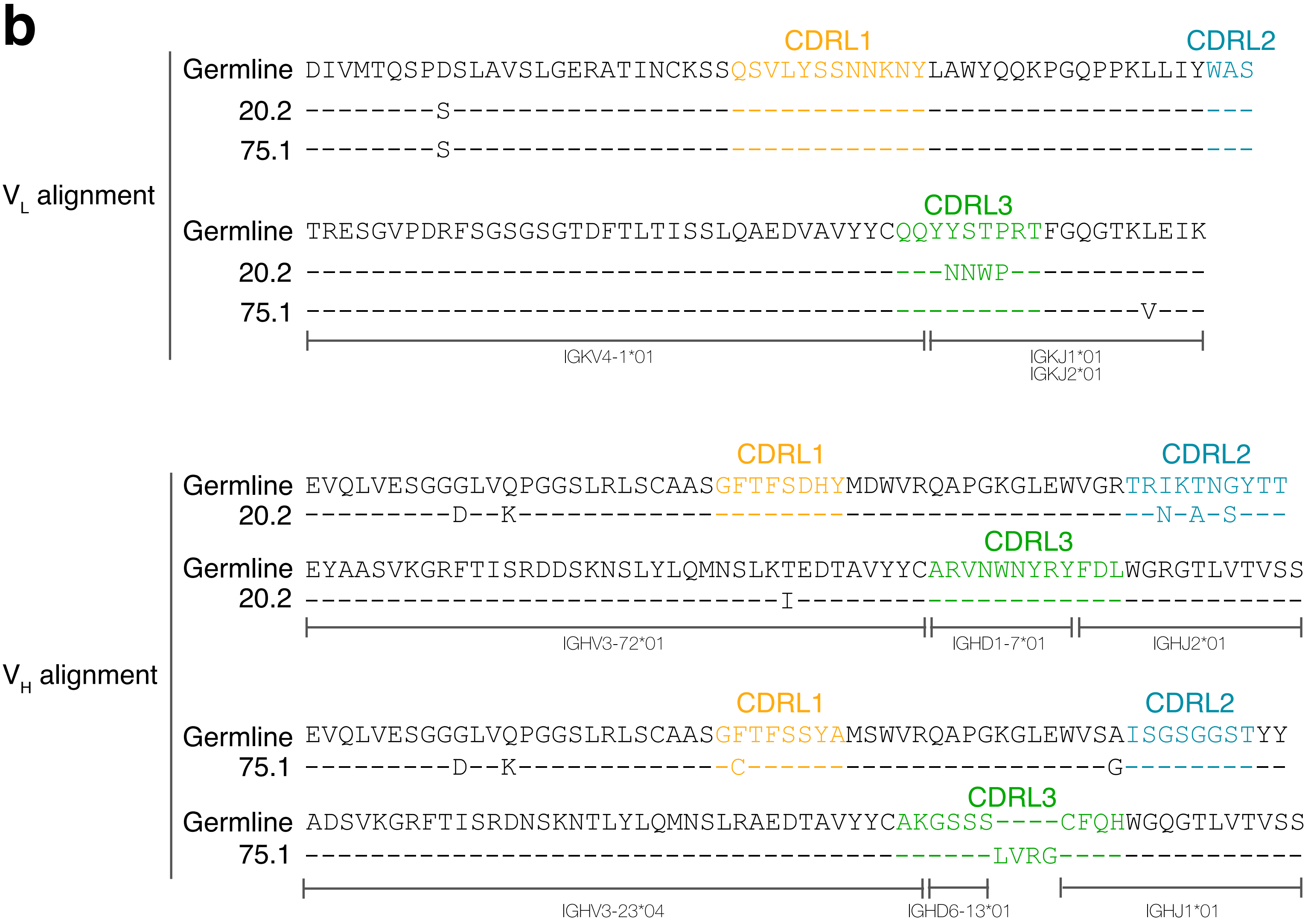

Supplementary Figure 3. Immunoglobulin variable domain sequence analysis by IgBLAST of cyclonal hits. (a) Germline gene usage and (b) assignment of 20-2 and 75-1 putative germline sequences using NCBI IgBLAST (Ye et al., 2013 *Nucleic Acids Research*).

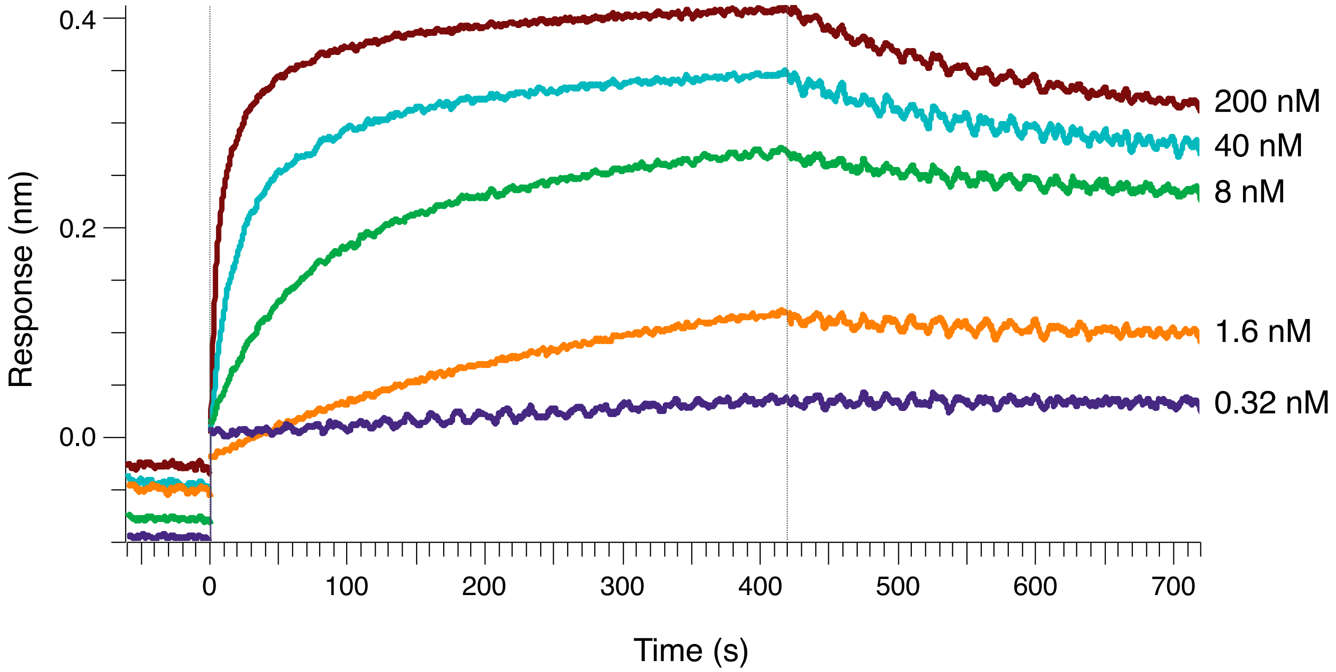

K_D = 7.457 +/- 0.065 x 10^{-10} (M)
k_a = 9.029 +/- 0.045 x 10^{5} (1/Ms)
k_d = 6.734 +/- 0.048 x 10^{-4} (1/s)
R^2 = 0.9901

Supplementary Figure 4. Binding analysis of anti-HAG cyclonal. Biolayer interferometry (BLI) affinity measurements for the anti-HAG cyclonal against GST-HAG. The cyclonal IgG was purified by protein A-based affinity capture. Response data are representative of replicate (n = 2) BLI experiments.
