# Supplementary material for: Isolation of full-length IgG antibodies from combinatorial libraries expressed in the cytoplasm of Escherichia coli
Source: Nat Commun. 2023 Jun 14;14:3514. doi: 10.1038/s41467-023-39178-x (PMC10267130; doi:10.1038/s41467-023-39178-x)
Supplement: Supplementary file 2 — Reporting Summary [file 41467_2023_39178_MOESM2_ESM.pdf]

nature portfolio

Corresponding author(s): Matthew DeLisa

Last updated by author(s): May 29, 2023

Reporting Summary

Nature Portfolio wishes to improve the reproducibility of the work that we publish. This form provides structure for consistency and transparency in reporting. For further information on Nature Portfolio policies, see our Editorial Policies and the Editorial Policy Checklist.

Statistics

For all statistical analyses, confirm that the following items are present in the figure legend, table legend, main text, or Methods section.

n/a	Confirmed	
☐	☒	The exact sample size (*n*) for each experimental group/condition, given as a discrete number and unit of measurement
☐	☒	A statement on whether measurements were taken from distinct samples or whether the same sample was measured repeatedly
☐	☒	The statistical test(s) used AND whether they are one- or two-sided<br>*Only common tests should be described solely by name; describe more complex techniques in the Methods section.*
☒	☐	A description of all covariates tested
☒	☐	A description of any assumptions or corrections, such as tests of normality and adjustment for multiple comparisons
☐	☒	A full description of the statistical parameters including central tendency (e.g. means) or other basic estimates (e.g. regression coefficient) AND variation (e.g. standard deviation) or associated estimates of uncertainty (e.g. confidence intervals)
☐	☒	For null hypothesis testing, the test statistic (e.g. *F*, *t*, *r*) with confidence intervals, effect sizes, degrees of freedom and *P* value noted<br>*Give P values as exact values whenever suitable.*
☒	☐	For Bayesian analysis, information on the choice of priors and Markov chain Monte Carlo settings
☒	☐	For hierarchical and complex designs, identification of the appropriate level for tests and full reporting of outcomes
☒	☐	Estimates of effect sizes (e.g. Cohen's *d*, Pearson's *r*), indicating how they were calculated

Our web collection on statistics for biologists contains articles on many of the points above.

Software and code

Policy information about availability of computer code

Data collection	Image Lab 6.1 software (Bio-Rad) was used for collecting spot plate images.
Data analysis	Prism 9 for macOS (version 9.2.0) was used for generating all graphs and performing all statistical analysis described in the manuscript. The kinetic data obtained using Octet RH16 instrument was analyzed using the Octet Analysis Studio 12.2.2.26 (Sartorious).

For manuscripts utilizing custom algorithms or software that are central to the research but not yet described in published literature, software must be made available to editors and reviewers. We strongly encourage code deposition in a community repository (e.g. GitHub). See the Nature Portfolio guidelines for submitting code & software for further information.

Data

Policy information about availability of data

All manuscripts must include a data availability statement. This statement should provide the following information, where applicable:

- Accession codes, unique identifiers, or web links for publicly available datasets
- A description of any restrictions on data availability
- For clinical datasets or third party data, please ensure that the statement adheres to our policy

All data generated or analyzed during this study are included in this article and its Supplementary Information/Source Data file. Source data are provided with this paper.

Human research participants

Policy information about studies involving human research participants and Sex and Gender in Research.

Reporting on sex and gender	N/A
Population characteristics	N/A
Recruitment	N/A
Ethics oversight	N/A

Note that full information on the approval of the study protocol must also be provided in the manuscript.

Field-specific reporting

Please select the one below that is the best fit for your research. If you are not sure, read the appropriate sections before making your selection.

[x] Life sciences [ ] Behavioural & social sciences [ ] Ecological, evolutionary & environmental sciences

For a reference copy of the document with all sections, see nature.com/documents/nr-reporting-summary-flat.pdf

Life sciences study design

All studies must disclose on these points even when the disclosure is negative.

Sample size	For experiments other than those involving mice, sample sizes were not predetermined based on statistical methods, but were chosen according to the standards of the field (at least three independent biological replicates for each condition), which gave sufficient statistics for the effect sizes of interest. In the specific case of Octet biolayer interferometry analysis, two biological replicates were performed. For the studies involving mice, library construction only required harvesting RNA from the spleen of a single mouse, which is consistent with the literature and is consistent with our research team's extensive work with antibody library construction.
Data exclusions	No data was excluded from the analyses in this work.
Replication	To verify the reproducibility of results from experiments other than those involving mice, we performed three biological replicates of each. In every experiment presented, the results were found to be reproducible. For the studies involving mice, library construction only required harvesting splenic RNA from one mouse and hence attempts at replication were not performed.
Randomization	The non-animal experiments were not randomized. All samples were analyzed equally with no sub-sampling and thus there was no requirement for randomization. Likewise, since only a single mouse was used to provide splenic RNA for DNA library construction, there was no requirement for randomization.
Blinding	For all experiments other than those involving animals, investigators were not blinded. Blinding during collection was not needed because conditions were well controlled. Blinding during analysis was not feasible as the differences between samples under different conditions were visually apparent in the collected data. Blinding is also not necessary because the results are quantitative and did not require subjective judgment or interpretation. Blinding is not typically used in the field. For animal experiments, investigators were not blinded. Blinding was not deemed necessary as only a single mouse was used to provide splenic RNA that was acquired indiscriminately by the experimenters and was subsequently used for construction of a synthetic DNA library by the experimenters following well-defined pre-established criteria.

Reporting for specific materials, systems and methods

We require information from authors about some types of materials, experimental systems and methods used in many studies. Here, indicate whether each material, system or method listed is relevant to your study. If you are not sure if a list item applies to your research, read the appropriate section before selecting a response.

Materials & experimental systems

n/a	Involved in the study	
[ ]	[x]	Antibodies
[x]	[ ]	Eukaryotic cell lines
[x]	[ ]	Palaeontology and archaeology
[ ]	[x]	Animals and other organisms
[x]	[ ]	Clinical data
[x]	[ ]	Dual use research of concern

Methods

n/a	Involved in the study	
[x]	[ ]	ChIP-seq
[x]	[ ]	Flow cytometry
[x]	[ ]	MRI-based neuroimaging

Antibodies

Antibodies used	rabbit anti-human IgG (Fc) antibody–HRP conjugate (ThermoFisher cat # 31423) [1:5,000-diluted]
Validation	The antibody from ThermoFisher is comprehensively validated for quality and performance (specificity,sensitivity, cross-reactivity) as discussed on their website (https://www.thermofisher.com/antibody/product/Rabbit-anti-Human-IgG-Fc-Secondary-Antibody-Polyclonal/31423). Detailed protocols for usage can also be found at these websites.

Animals and other research organisms

Policy information about studies involving animals; ARRIVE guidelines recommended for reporting animal research, and Sex and Gender in Research

Laboratory animals	Humanized ATX-GK mice with complete functional human gamma heavy chain and kappa light chain on a BL/6 background (MHC Haplotype H-2b) were obtained from Alloy Therapeutics. Mice were housed under the following environmental conditions to reduce stress: 14-hour light/10-hour dark cycle and temperature of ~70F with ~50% humidity.
Wild animals	No wild animals were used in the study.
Reporting on sex	Both male and female mice were purchased with splenic RNA used in library construction obtained from a randomly chosen female mouse.
Field-collected samples	No field-collected samples were used in the study.
Ethics oversight	The protocol number for the animal trial was 2012-0132 and was approved by the Institutional Animal Care and Use Committee (IACUC) at Cornell University.

Note that full information on the approval of the study protocol must also be provided in the manuscript.
