# Supplementary material for: Isolation of full-length IgG antibodies from combinatorial libraries expressed in the cytoplasm of Escherichia coli
Source: Nat Commun. 2023 Jun 14;14:3514. doi: 10.1038/s41467-023-39178-x (PMC10267130; doi:10.1038/s41467-023-39178-x)
Supplement: Supplementary file 3 — Description of Additional Supplementary Files [file 41467_2023_39178_MOESM3_ESM.pdf]

Title: Supplementary Dataset 1
Description: Excel spreadsheet with primers used in library construction
